# ADVANCING BRAIN HEALTH THROUGH THE DATA FOR ACTION PROJECT

**DOI:** 10.1093/geroni/igae098.1557

**Published:** 2024-12-31

**Authors:** Meghan Fadel, Tyrone Bethune, Talyah Sands, Shelby Roberts, Erin Bouldin, Amanda Cheney

**Affiliations:** Alzheimer’s Association, Chicago, Illinois, United States; Association of State and territorial health officials, Arlington, Virginia, United States; Association of State and Territorial Health Officials, Arlington, Virginia, United States; Alzheimer’s Association, Chicago, Illinois, United States; University of Utah, Salt Lake City, Utah, United States; University of Utah, Orem, Utah, United States

## Abstract

The Healthy Brain Initiative (HBI) Data for Action Project is a nationwide effort to support the integration of data on brain health and caregiving into public health planning efforts. Data about cognitive health, dementia, and family caregiving are increasingly available at the state level, in part due to HBI efforts to support the implementation of the Behavioral Risk Factor Surveillance System (BRFSS) optional modules on Cognitive Decline and Caregiving. This effort has increased state implementation of the Cognitive Decline Module from 5 states in 2009 to 49 states and territories in 2019-2020. Similarly, the Caregiver Module implementation grew from 4 states in 2009 to 49 states and territories in the years 2021-2022. The Alzheimer’s Association, alongside partners at the Association for State and Territorial Health Officials (ASTHO), developed the Data for Action Project in 2023 to support state health departments as they use public health data to advance brain health in their communities. In its first year, 4 states participated in the Data for Action pilot by conducting data analyses, producing dissemination tools of the findings, and creating a strategic plan to share the results. States participated in regular cohort calls, provided one another feedback, received individualized technical assistance, and had opportunities to connect through an asynchronous platform. The Data for Action Project created a community of practice with education, peer support and technical assistance for participating health departments.

